# Translational perspectives to treat Epidermolysis bullosa—Where do we stand?

**DOI:** 10.1111/exd.14194

**Published:** 2020-12-02

**Authors:** Christine Prodinger, Johann W Bauer, Martin Laimer

**Affiliations:** ^1^ Department of Dermatology and Allergology University Hospital of the Paracelsus Medical University Salzburg Salzburg Austria

**Keywords:** barrier disruption, gene therapy, genodermatoses, immunomodulation, pathogenesis, targeted therapy

## Abstract

Epidermolysis bullosa (EB) is the prototypical example of genetic skin fragility disorders. Genotypic heterogeneity, modifier genes, epigenetic, biochemical and environmental factors alter and determine pathogenic traits and, ultimately, the wide and striking phenotypic variability in EB. Besides the primary structural‐functional defect, chronic tissue damage with induction and dysregulation of inflammatory pathways is a common pathogenic mechanism in EB. In localized variants, the inflammatory aberrations may mainly affect the micromilieu of lesional skin, while a systemic inflammatory response was shown to contribute to the systemic morbidity in severe EB subtypes with extensive cutaneous involvement. Our continued understanding of the pathophysiology of EB, as well as advances in molecular technologies, has paved the way for translational therapeutic approaches. The spectrum comprises of corrective and symptom‐relieving therapies that include innovative therapeutic options garnered from the bench, repurposed drugs approved for other diseases, as well as strategies for gene‐, protein‐ and cell‐based therapies. Immunological traits further define new targets of therapy, aimed at improving skin barrier restoration, microbial surveillance and infection control, wound healing and anti‐neoplastic effects. Clinical availability and feasibility of these approaches for all EB patients and subtypes are currently limited, reflecting issues of efficacy, specificity, tolerability and safety. A multistep targeting approach and highly individualized, risk‐stratified combinatory treatment plans will thus be essential for sustained efficacy and improved overall quality of life in EB.

## 1 INTRODUCTION

Epidermolysis bullosa (EB) comprises a group of rare, genetically determined skin fragility disorders characterized by (muco‐) cutaneous blistering following mild mechanical trauma. The most recent classification guidelines published in February 2020 define EB as the prototypic genetic disorder of skin fragility. Other skin fragility disorders, including peeling skin disorders, erosive disorders, hyperkeratotic disorders and connective tissue disorders with skin fragility, are now classified as a separate category, that is so‐called “EB‐related disorders.”^[^ ^1^ ^]^ Blistering constitutes only a minor aspect of clinical presentation in these entities, or even not at all, owing to the very superficial skin cleavage.

Currently, mutations in 16 different genes have been implicated in the pathogenesis of classical EB.^[^ ^1^ ^]^ These genes encode proteins essential for sustaining structural and functional integrity of the epidermis and dermo‐epidermal basement membrane zone, a highly specialized interface between epithelial cells and the underlying matrix that is crucial for cell adhesion, proliferation and differentiation, tissue repair and barrier function. Expression of EB index genes in other epithelialized (gastrointestinal, respiratory and urogenital tract) or mesenchymal (skeletal muscle) tissues accounts for a primary multi‐organ involvement that, especially in severe subtypes, causes significant morbidity and mortality.^[^ ^1^, ^2^, ^3^ ^]^

Besides a considerable genotypic heterogeneity, modifier genes, epigenetic and biochemical, as well as environmental factors alter and determine pathogenic traits and, subsequently, the wide phenotypic variability in this group of disorders. Examples include *in cis* gene variants that change the expression of the corresponding allele, postzygotic or revertant mosaicism, variants in genes whose products modulate or influence EB‐associated proteins, differential regulation of several other genes involved in the maintenance and function of this microenvironment, as well as induction of inflammatory cascades following genetically determined tissue injury.^[^ ^1^, ^4^ ^]^

The mainstay of current clinical management is on protection and avoidance of provoking factors, as well as palliation of cutaneous disease manifestations. Therapeutic principles aim at wound care and improved healing, management of microbial burden and infection, control of pain and itch, as well as prevention and supportive care for complications, like anaemia, malnutrition and skin cancer.^[^ ^5^, ^6^ ^]^ In light of the serious morbidity and mortality of EB, these primarily symptom‐orientated and supportive treatment strategies do not sufficiently fulfil the medical needs of a critical proportion of patients.

An improved understanding of the pathophysiology of EB, together with advances in molecular technologies, is paving the way for translational therapeutic approaches. The spectrum comprises of corrective and symptom‐relieving therapies, including innovative therapeutic components from the bench, repurposed drugs approved for other (more common) diseases like cancers or immune disorders, as well as strategies for gene‐, protein‐, cell‐based and small molecule therapies.^[^ ^7^, ^8^, ^9^ ^]^

## 2 LOOKING BACK IN ORDER TO MOVE FORWARD—LESSONS LEARNED ON CHALLENGES OF TESTING THERAPIES IN EB

The number of investigational products tested in clinical trials for their translational therapeutic potential in EB is constantly growing (Table 1). Clinical research in EB offers promising perspectives but also faces relevant methodological challenges, of which most are inherent to studies conducted in rare disease populations.^[^ 10, 11, 12

- First, intrinsically low patient numbers and recruitment failures impair sample size requirements and compromise statistical power while increasing trial duration and costs. As such, slight or moderate changes in response to treatment hardly reach statistical significance when using standard methods (limited acceptable evidence of efficacy). Sample size calculation is often difficult, due to limited information about variances and correlations in the planning phase (because of lack of reliable pilot studies), and failure of classical statistical approaches that are either based on large‐sample approximations (ie asymptotic arguments), or rely on distributional assumptions that cannot easily be specified in small sample numbers. Moreover, ethical concerns regarding the use of placebo in severe cases, as well as conducting research in children have to be weighed against the advantages afforded by a randomized controlled study design.
- A still incomplete understanding of pathogenic disease traits, natural course and potential therapeutic targets (or mechanisms of intervention) further causes difficulties in determining key milestones, suitable clinical trial endpoints, appropriate study length, accurate effect size, as well as types and timing of validated outcome measures. Inappropriate timing might increase the number of drop‐outs.
- The heterogeneity of EB and diseased study cohort is reflected by many geno‐/phenotypes, inconsistent genotype‐phenotype correlations, improper diagnostics and soft inclusion criteria to foster recruitment. These characteristics inherently introduce an enhanced degree of random covariate imbalance in small samples that might limit generalizability and applicability of trial results to real‐life settings.
- An increasing trial complexity due to methodological, logistical and regulatory challenges critically impairs trial feasibility. For instance, the number of subjects that are both eligible and inclined to participate based on disease profiles and health status is typically small and candidates are frequently geographically dispersed. This impedes the gathering of a sufficient sample size within reasonable timeframes and costs to support hypothesis testing and characterisation of interventional benefits and risks. In multi‐centre trial settings intended to increase sample size through (international) recruitment, complex endpoints that require additional special handling or expertise may further reduce the number of study sites able or inclined to participate.

**TABLE 1:** Emerging and ongoing clinical trials and therapeutic approaches for Epidermolysis bullosa^[^ 9, 22

Therapeutic approach	Trial characteristics	EB subtype	Trial identification number/Reference
Ex vivo gene replacement therapy			
Grafting of epidermal sheets containing epidermal stem cells, gene‐corrected with a gamma‐retroviral vector carrying *COL17A1* cDNA	Phase I/II; HOLOGENE17	JEB	NCT03490331
Transplantation of *COL7A1*‐corrected autologous keratinocyte sheets (EB‐101)	Phase III; VIITAL	RDEB	NCT04227106
Transplantation of *COL7A1*‐SIN retroviral engineered autologous tissue‐engineered skin	Phase I/II; GENEGRAFT	RDEB	NCT04186650
In vivo gene replacement therapy			
Topical gel of non‐integrating, replication‐deficient HSV‐1 vector containing two functional *COL7A1* genes (KB103)	Phase II	RDEB	NCT03536143
Synthetic polymer polyplexes containing *COL7A1*, for topical application (AP103)	Phase I	RDEB	https://www.amrytpharma.com/patients‐and‐carers/gene‐therapy/
Gene/Cell therapy			
*COL7A1*‐genetically modified autologous fibroblasts injected in the wounds (FCX‐007)	Phase III	RDEB	NCT04213261
Allogeneic ABCB5+ (mesenchymal) stem cells (systemic infusion)	Phase I/II	RDEB	NCT03529877
Haploidentical MSCs derived from bone marrow (systemic infusion)	Phase I/II	RDEB	NCT04153630
Allogeneic hematopoietic stem cell transplantation and serial donor or “off‐the‐shelf” MSCs infusions	Phase II Phase II	Severe EB Severe EB	NCT02582775 NCT01033552
Systemic allogeneic mesenchymal stromal cells	Phase I	RDEB	[16]
Gene editing approach			
Genomic editing via CRISPR/Cas9 nuclease system	Preclinical	RDEB, JEB, EBS	[82]
RNA‐based therapy			
Antisense oligonucleotide Q3‐313 for topical application in DEB patients with pathogenic mutation(s) in exon 73 in *COL7A1* gene	Phase I/II	DEB	NCT03605069
Trans‐splicing approaches	Preclinical	RDEB, EBS	[91]
Protein therapy			
Recombinant human collagen VII (PTR‐01)	Phase I/II	RDEB	NCT03752905
Small molecules/biologics			
Topical and intravenous *Gentamicin*, a PTC read‐through for restoration of functional protein in patients with nonsense mutations	Phase I/II Phase I/II Phase I/II	JEB JEB RDEB	NCT03526159 NCT04140786 NCT03392909
*HMGB1* peptide drug, regeneration‐inducing	Phase II	RDEB	[16]
Anti‐inflammatory ointment *Diacerein 1%*	Phase II	EBS	NCT03389308
*Oleogel‐S10* for wound healing	Phase III	EB	NCT03068780
*Thymosin‐β4 (RGN‐137)* for wound healing	Phase II	JEB, DEB	NCT03578029
*Coenzyme Q10* (BPM31510 3%) cream for wound healing	Phase I	EB	NCT02793960
*Botulinic toxin* injections in plantar lesions for clinical improvement	Phase II/III	EBS	NCT03453632
Topical analgesic *Ropivacaine*	Phase II	EB	NCT03730584
*Pregabalin* for neuropathic pain and pruritus	Phase III	RDEB	NCT03928093
Neurokinin‐1 receptor antagonist *Serlopitant* for pruritus	Phase II	EB	NCT03836001
*Rigosertib* for advanced squamous cell carcinoma	Phase I Phase I/II	RDEB RDEB	NCT04177498 NCT03786237
PD‐1 inhibitors (eg *Nivolumab*) for advanced squamous cell carcinoma	Phase II Phase II	RDEB RDEB	NCT04204837 NCT03834233

Approaches to overcome these hurdles comprise, for example, a close collaboration between sponsor, academia, regulatory agencies and patient groups to encourage a patient‐centric trial design. This means involving affected individuals and their caregivers in decisions on study length and portfolio, target population, in‐/exclusion criteria, definition of clinically meaningful endpoints, appropriate timing of outcome assessments, as well as information policies, including timely disclosure of trial results. Patient‐centricity aims at reducing the patients‘ trial burden, for example in terms of time, travel, number of scheduled visits, literacy, personal financial expenditures, and compatibility with occupational obligations, and importantly, extent of invasive investigations on highly fragile, chronically wounded skin. Patient engagement would also likely optimize trial recruiting and reduce the number of drop‐outs. Faster enrolment and improved compliance subsequently help to reduce expenses in inherently cost‐sensitive rare disease research.^[^ 10, 13

Properly trained clinical study teams are required to ensure quality and professionalism. Likewise, global strategies are needed to navigate and satisfy the requirements and policies set forth by different regulatory bodies, funding agencies and participating institutes, if we are to advance the EB clinical research agenda via international networks/consortia.

Another key strategy in EB trials is to make use of the limited available information as efficiently as possible. This includes, for example, usage of multiple/composite endpoints and multiple treatment arms, incorporation of interim data review, or formal synthesis of previously collected data (eg Bayesian statistical methods). Likewise, alternative clinical trial designs (such as series of n‐of‐1 trials design, response‐adaptive study design, randomized withdrawal design; factorial designs) hold some promise to increase trial acceptability, optimize randomization procedures and mitigate effects of clinical heterogeneity, as well as to decrease sample size requirements by applying statistical methods to adapt the significance level in small populations.^[^ 10, 14, 15

In this context, natural history studies and registries of well‐described patient cohorts enable essential insight into disease mechanisms and characteristics, which can be leveraged to define surrogate markers or prognostic indicators, as well as clinically meaningful endpoints. Examples include the International EB Registry, PEBLES (Prospective Epidermolysis Bullosa Longitudinal Evaluation Study) and the EB Clinical Characterization and Outcomes Database (CCOD).^[^ ^16^ ^]^

## 3 LOOKING AT THE STATUS QUO—ADVANCED THERAPEUTIC MODALITIES FOR EB

Progress in molecular research has enhanced our knowledge about pathogenic traits in EB, thereby providing new therapeutic insight. The number of innovative treatment modalities, including causal approaches as well as symptom‐relieving therapies, currently tested in clinical trials, is steadily growing.^[^ 7, 9, 17

## 4 SYMPTOM‐RELIEVING AND DISEASE‐MODIFYING THERAPIES

It has become evident that apart from the primary structural‐functional defect, chronic tissue damage with induction and dysregulation of inflammatory pathways is a common pathogenic mechanism in EB. In severe subtypes such as recessive dystrophic EB (RDEB), extensive cutaneous involvement is implicated to further have a systemic inflammatory impact that contributes to the morbidity.^[^ 18, 19, 20, 21 Deeper insight into pathogenic traits have enabled the delineation of putative therapeutic targets. Some of these targets may be mechanistically addressed by (repurposing of) readily available drugs used for other disease entities. Clinical validation of such palliative, so‐called symptom‐relief and disease‐modifying therapies in the complex setting of EB, however, is essential. The implementation of these strategies still poses several challenges, including technical and financial issues, as well as concerns about feasibility, safety and sustainability of effects.^[^ ^22^ ^]^ Against this background, a considerable number of therapeutic strategies, focusing on aberrant molecular and immune‐regulatory traits involved in pain, itch, protracted tissue damage and chronic wounding as well as secondary disease sequelae like inflammation, scarring and squamous cell carcinoma, have reached a clinical trial stage (Table 1).

In severe EB simplex, blistering has been linked to an aggregation of mutated keratins (5/14) within basal keratinocytes after infectious, physical or chemical stress.^[^ ^23^ ^]^ Subsequent inflammatory stress signatures comprise uncontrolled Th17 activation and enhanced maturation of pro‐inflammatory cytokines like IL‐8, IL‐1β and IL‐5, ultimately leading to epidermal apoptosis.^[^ 24, 25 Consistently, apremilast, a systemic PDE‐4 inhibitor approved for psoriasis that impairs Th1/Th17 activation, recently demonstrated clinical efficacy in a small study, decreasing blistering in three EBS patients with high levels of T helper 17 cytokines in lesional skin.^[^ 24, 26

Likewise, diacerein, a small molecule derived from the rhubarb root that inhibits pro‐inflammatory IL‐1β signalling and the c‐jun N‐terminal‐kinase (JNK) stress pathway, was shown to significantly reduce blister numbers when applied topically in *KRT5* or *KRT14* mutated severe EBS patients.^[^ 27, 28 After investigator‐driven trials, additional studies are ongoing which focus on pharmacokinetics after maximum or long‐term use. (NCT03389308).

Topical calcipotriol, an active vitamin D3 analogue, is known to have immunomodulatory properties by increasing expression of cathelicidin and enhancing antimicrobial defense.^[^ ^29^ ^]^ Interim results of a double‐blind, placebo‐controlled crossover study on 9 patients with RDEB, revealed that low‐dose topical calcipotriol (0.05mg/g in Ultraphil®) significantly reduced pruritus and induced transient improved wound healing.^[^ 18, 30

Repetitive injury, skin blistering and wounding are main causes of tissue remodelling and progressive scarring, leading to joint contractures and mitten deformities of hands and feet, particularly in RDEB. The chronic inflammatory state also enhances the risk of malignant transformation and development of squamous cell carcinomas. Based on preclinical data identifying TGFβ produced by RDEB fibroblasts to be a key player in mediating injury‐driven inflammation and secondary fibrosis, a phase I/II clinical trial assessing the TGFβ‐inhibitor losartan was initiated 2017. Interim analysis of 18 children aged 3‐16 years revealed good tolerability and safety, as well as a promising impact on fibrotic and systemic inflammatory markers. Based on this data, a phase II/III trial is currently in preparation.^[^ ^31^ ^]^

Emerging and ongoing clinical trials and therapeutic approaches for Epidermolysis dupilumab, a monoclonal antibody approved for atopic dermatitis that targets IL‐4Rα,showed clinical efficacy in a 52‐year‐old patient with a highly pruriginous subtype of EB (EB pruriginosa).^[^ 33 ^]^ Dupilumab led to a rapid and significant clinical improvement of disabling itch and prurigo‐like skin lesions, although its impact on pruritogenic and inflammatory pathways in EB remains to be determined.^[^ 33, 34, 35 Chronic pruritus constitutes a major individual complaint in many EB patients, often inducing a vicious itch‐scratch‐blister cycle that is frequently inadequately controlled and resistant to treatments.^[^ ^36^ ^]^ Its intensity correlates with the severity of the EB subtype.^[^ 36, 37 Notably, *Staphylococcus aureus*, colonizing more than 90% of chronic wounds in EB, is able to induce upregulation of pro‐inflammatory cytokines such as TSLP, IL‐4, IL‐12 and IL‐22 and stimulates mast cell degranulation, resulting in Th2 skewing with skin inflammation and activation of sensory dorsal root ganglia neurons.^[^ 38, 39, 40, 41

In addition, regenerative cell therapies are clinically tested for favourable immunomodulatory effects. Allogeneic hematopoietic stem cell transplantation (HSCT) is a systemic disease‐modifying and symptom‐relieving approach. HSCT has so far demonstrated partial amelioration of the disease phenotype in patients with RDEB, including increased basement membrane integrity and (temporarily) reduced skin blistering.^[^ ^42^ ^]^ However, even with minimum intensity conditioning protocols, morbidity and mortality still remain high (10%‐15%).^[^ 43, 44 Addition of mesenchymal stem cell (MSC) infusions is currently being evaluated for an immunosuppressive effect, potentially allowing for a further attenuated conditioning regimen and lower peri‐interventional risks of immunomyeloablation.^[^ 45, 46 (NCT02582775, NCT01033552) Apart from enhancing the safety and efficacy of the HSCT, MSCs may also serve as an additional source of renewable cells for the treatment of focal areas of residual blistering.^[^ 47, 48, 49

Moreover, infusion of allogeneic bone marrow‐derived ABCB5+ (ATP‐binding cassette subfamily B member 5) dermal MSC, a subtype with increased potency to modulate underlying inflammatory response in a RDEB mouse model, is currently being tested in a phase 1/2 clinical trial in RDEB patients. (NCT03529877).^[^ 50, 51

Accumulation of bone marrow‐derived circulating mesenchymal stem cells in injured skin was recently shown to be stimulated by the HMGB1 peptide drug in a murine model of dystrophic EB model. Consequently, inflammation was suppressed and regeneration of both mesenchymal and epidermal components promoted. A phase II trial with RDEB patients is currently aiming to corroborate this preclinical observation.^[^ 16, 52

Although providing evidence for more distinctive targeting than classical immunosuppressive medications (like glucocorticoids, cyclosporine and methotrexate), these immunomodulatory agents, particularly when administered systemically, still harbour the risk of adversely compromising (distinct aspects of) the host defense. While the connection between the immune system and distinct subtypes of EB remains to be determined, patients are not known to have any severe immunodeficiency.^[^ 53, 54, 55 On the other hand, extensive cutaneous, mucosal and organ involvement increases the risk for infections and skin tumors in the severe types. This renders patient compliance and regular follow‐up critical.

## 5 TARGETED ANTI‐TUMOR THERAPY IN EB

Squamous cell carcinoma (SCC) of the skin is a frequent complication, particularly in RDEB. It arises already in early adulthood and often develops at sites of chronic wounds, regeneration or scarring. The course is aggressive with high rates of recurrence and metastasis. RDEB SCCs are usually very poorly responsive to conventional chemotherapy agents and radiotherapy, making SCC the leading cause of death of several subtypes of EB.^[^ 3, 56 Suggested pathogenic mechanisms underlying the tumor propensity comprise repetitive tissue stress and remodelling with injury‐driven induction of inflammatory and tumor‐promoting pathways. These include growth activation of keratinocytes, polymorphisms in matrix metalloproteinases, and compromised immune surveillance with reduced activity of natural killer cells.^[^ 57, 58, 59, 60, 61, 62 The chronic inflammatory state, with lead mediators in RDEB including transforming growth factor‐β (TGF‐ß), tumor necrosis factor α (TNF‐α) and IL‐6, is implicated in the biophysically altered, fibrotic tissue microarchitecture, which is characterized by matrix stiffening, aberrations of mechano‐signalling and enhanced epithelial‐to‐mesenchymal transition in keratinocytes. Such alterations are proposed to favour a pro‐inflammatory, tumorigenic micromilieu, for example through enhanced migratory capacity, invasiveness and elevated resistance to apoptosis of keratinocytes.^[^ 18, 53, 58, 63, 64, 65, 66, 67, 68 The presence of flagellated bacteria promoting innate sensing may be an additional tumor‐promoting factor.^[^ 69, 70

Clinical trials are currently underway to assess anti‐tumor activity of rigosertib (NCT03786237, NCT04177498), a serine/threonine‐protein kinase (Polo‐like Kinase 1) inhibitor that leads to apoptosis specifically in RDEB cancer cells,^[^ ^71^ ^]^ as well as of the programmed cell death protein 1 (PD‐1) inhibitors nivolumab and cemiplimab.^[^ ^72^ ^]^ While the latter have become a standard treatment for advanced non‐EB SCC, administration in EB patients is only anecdotally reported.^[^ 73, 74, 75 Thus, their therapeutic and immunomodulatory impact on EB hitherto remains largely unknown, including potentially disadvantageous effects on barrier integrity, local and systemic inflammatory state, microbial burden and susceptibility to skin infections. In addition, common autoimmune skin (eg rash and pruritus) and gastrointestinal (eg diarrhoea) toxicities (may be challenging for critical stage IV tumor patients with dystrophy and severe cutaneous involvement, which is characteristic of RDEB phenotypes.^[^ ^76^ ^]^

## 6 TREATMENT OPTIONS WITH CURATIVE POTENTIAL

Apart from disease‐modifying therapeutic modalities, diverse translational research strategies are moving into pivotal phase 3 trials, aiming at correction of the genetic defect or substitution of the dysfunctional components at the DNA, mRNA, protein and cellular level.

*Protein replacement therapy* with PTR‐01, a recombinant human collagen 7 (C7) intravenously administered in multiple ascending doses, is currently investigated in a clinical phase I/II trial for safety and tolerability. (NCT03752905) Protein size and a tendency to form aggregates, however, limit skin homing, as well as accessibility to other extracutaneous, EB‐affected tissues, where it is needed to aid repair, thereby compromising the clinical feasibility of this therapeutic option.^[^ 77, 78

*Cell therapies* under investigation include intradermal injections of genetically modified, C7‐overexpressing, patient‐autologous human dermal fibroblasts (FCX‐007), which have the potential for increased and prolonged clinical impact. A phase 1/2 clinical trial for RDEB is ongoing (NCT02810951) to evaluate C7 expression, anchoring fibril formation, as well as evidence of wound healing. Interim results indicate good tolerance and a sustained efficacy compared with control during intervention.^[^ ^79^ ^]^

Induced pluripotent stem cells (iPSCs) are artificial stem cells derived from transduced and reprogrammed somatic cells that can be differentiated into diverse cell types, including keratinocytes and fibroblasts. Their autologous nature reduces the risk of immune rejection of the corresponding cell‐expanded tissue grafts. At the preclinical level, strategies for gene‐correction of disease‐specific iPSCs have been demonstrated. CRISPR/Cas9‐mediated homologous recombination (HR) to correct a splice‐site mutation in *COL7A1* was combined with the piggyBac transposon system to remove residual gene fragments inserted by gene editing. This strategy achieved footprint‐free genomic repair with improved efficiencies, while maintaining low adverse repair outcomes.^[^ ^80^ ^]^ Moreover, successful generation and application of fully autologous skin equivalents was recently shown in an animal model using CRISPR/Cas9‐corrected keratinocytes and fibroblasts differentiated from iPSCs.^[^ ^81^ ^]^ Although gene editing strategies can be easily developed for most EB‐associated mutations (including dominant negative aberrations), the applicability of this technique is still restricted by regulatory concerns over safety (off‐target effects), efficacy and quality control.^[^ ^82^ ^]^ To increase both editing efficiency and specificity, current developments focus on, for example optimizing repair templates, their delivery as ribonucleoprotein complexes by electroporation, exon reframing approaches for frameshift‐inducing indel mutations or prime editing systems.^[^ ^82^, ^83^ The latter use a catalytically impaired Cas9 fused to an engineered reverse transcriptase domain that, when programmed with a prime editing guide RNA (pegRNA), is capable of directly inserting the desired edits into a specific DNA site with single nucleotide precision without the need for double‐strand breaks or exogenous HR templates.^[^ ^84^ ^]^

*Premature termination codon (PTC) read‐through therapies* aim to restore expression of full‐length protein, by enabling the incorporation of an amino acid at a premature stop codon, rather than terminating ribosomal translation.^[^ ^85^ ^]^ Gentamicin, an aminoglycoside antibiotic, was shown to induce PTC read‐through and proved efficacious to varying degrees in clinical trials with patients harbouring *COL7A1, COL17A1* and *LAMB3* PTC mutations, when administered both topically and intravenously.^[^ ^86^, ^87^, ^88^, ^89^ While PTC mutations are common in most EB genes, efficacy of PTC read‐through depends on the type of stop codon, its sequence, as well as neighbouring nucleotides, which currently narrows down its broad applicability.^[^ ^90^ ^]^ Moreover, while topical gentamicin is considered a convenient modality (that is easily administered and readily prescribed as being commercially available, inexpensive and safe), systemic application is limited due to nephro‐and ototoxic effects.^[^ ^85^ ^]^ Two follow‐up studies (NCT03392909) (NCT04140786) are currently investigating the tolerability, safety and efficacy of intravenous gentamicin.

*Antisense oligonucleotides* (AONs) are small stretches of modified DNA or RNA that can be utilized to induce skipping of mutation‐containing, in‐frame, non‐essential exons during the transcription process. The resulting alternate mRNA produces a shortened but ideally functional version of the protein. AONs are usually rather easy to manufacture and administer, have low toxicity and cause limited adverse events. A phase 1/2 multicenter clinical trial applying a topical formulation of AON (Q313) in DEB patients is currently ongoing (NCT03605069). Participants must harbour mutation(s) in exon 73 of the *COL7A1* gene, whose AON‐mediated in‐frame deletion is unlikely to cause major structural changes in the affected protein.^[^ ^91^, ^92^

*Gene replacement therapy* replaces a non‐functioning gene with a synthetic copy of a functional gene. This therapeutic modality has entered clinical development, employing transposons, retroviral or lentiviral vectors (Table 1).^[^ ^7^, ^8^, ^93^, ^94^, ^95^

Ex vivo gene delivery approaches have proven to give rise to long‐lasting, biomechanically sound grafts in junctional EB patients with *LAMB3* mutations.^[^ ^32^, ^96^, ^97^, ^98^ However, technological issues relating to vector safety (ie risk of insertional mutagenesis), optimal delivery (especially of large genes like *COL7A1*), identification and targeting of holoclone stem cells (ie long‐lived epidermal stem cells with high colony‐forming efficiency^[^ ^99^ ^]^), as well as transfection/transduction efficiency to reach stable and controlled integration and activity of the transgene, hitherto limit the availability and long‐term effects of such treatments.^[^ ^8^ ^]^ Additionally, gene replacement has been shown in some cases to result in differential adhesive phenotypes between the transduced (corrected) and non‐transduced (mutant, wildtype) cells, potentially conferring a selective advantage to one over the other during in vitro culture expansion and sheet production, that might ultimately impact the robustness, long‐term homeostasis and regenerative capacity of the epidermal grafts.^[^ ^8^, ^100^ Apart from this, the interventional burden of the transplantation to the patient, as well as our limited understanding of potential autoimmune phenomena against therapeutically introduced neoantigens that might impact maintenance of the graft, also requires consideration. For dystrophic EB types, strategies for *COL7A1* correction may further necessitate targeting of both C7‐synthesizing cells, that is keratinocytes and fibroblast, for optimal assembly of skin anchoring fibrils as it has been shown in preclinical models.^[^ ^101^ ^]^

Recent approaches of in vivo gene therapies use a modified replication‐deficient and non‐integrating, epidermotropic herpes simplex type 1 virus to deliver therapeutic genes. Repetitive administration of this vector was found to be safe and effective in melanoma clinical trials.^[^ ^102^ ^]^ In a phase 1/2 study, topical application of beremagene geperpavec (B‐VEC), containing two functional *COL7A1* genes, is currently being investigated, and preliminary results reveal enhanced wound closure in patients. This data will be corroborated in an upcoming phase 3 study.^[^ ^103^ ^]^ As the transgene does not integrate into the recipient's genome, repetitive application is likely necessary to achieve sustained responses.

## 7 LOOKING INTO THE NEAR FUTURE OF EB THERAPY: SEEKING ORIENTATION IN INNOVATION

Curative treatment approaches for sustained re‐expression of a corrected gene still await broad clinical availability and might even not be applicable for all patients and subtypes due to current limitations in terms of feasibility, efficacy, specificity and safety. Symptomatic approaches have shown the potential to prevent, alleviate and treat cutaneous, mucosal and systemic symptoms and their sequelae/complications. Any of these principal approaches have to accurately address issues of safety, tolerability, feasibility and efficacy as well as cost‐effectiveness.^[^ ^15^ ^]^ In this context, a multistep targeting approach and highly individualized, risk‐stratified combinatory treatment plans will be essential for sustained efficacy and improved overall quality of life in EB.

In the near future, such an approach may involve current care standards in addition to

- gene replacement therapies, with either transplantation of sheets or topical application of emulgated/dispersed molecularly corrected cells/tissue (or corrective agents) onto sites of chronic wounding;
- topical applications, which offer vulnerable EB cohorts increased tolerability and feasibility and are of particular value for treating mucosal lesions and poorly accessible skin areas that are infeasible or challenging for skin grafting, like interdigital/intertriginous areas and face;
- skin grafting that is mainly restricted to sites of chronic and/or large blistering erosions to improve quality of life by reduction of itch, pain, inflammation, body fluid and protein loss while strengthening microbial defense and preventing cancer development in high‐risk long‐standing wounds;
- systemic and local immune modulation, for example iv stem cell infusions or ideally oral or liquid maintenance (immunomodulatory) therapies as booster, and topical agents like diacerein, calcipotriol;
- symptom‐relieving therapies, targeting distinct symptoms such as scarring (TGF‐β, TSP1^[^ ^104^ ^]^ and losartan^[^ ^31^, ^105^), itch (dupilumab^[^ ^31^ ^]^) and skin cancer (rigosertib,^[^ ^7^ ^]^ pembrolizumab^[^ ^72^ ^]^ and nivolumab (EudraCT 2016‐002811‐16));
- strategies for microbial surveillance (eg glucose peroxidase‐lactoperoxidase gel; hydrofiber dressings; quorum sensing),^[^ ^15^ ^]^ and
- last but not least, various combinations of these approaches, applied in a complementary or synergistic fashion.

## 8 PIPELINE OF OPTIMISM

Investigations to evaluate the regenerative therapeutic potential of MSC‐derived exosomes for various intractable inflammatory diseases, including EB, are stated to start this year (NCT04173650). These extracellular vesicles are key paracrine effectors of MSCs that carry anti‐inflammatory components (therapeutic miRNAs, mRNAs, cytokines, lipids and growth factors) for delivery to the target cells.^[^ ^106^, ^107^ Avoidance of injection of live cells suggests a favourable safety profile.

Recent preclinical research activities have identified some agents with promising translational potential, including inhibition of the APOBEC (apolipoprotein B mRNA editing enzyme, catalytic polypeptide‐like) family of enzymes. Their enhanced activity in response to environmental factors (such as skin‐injury dependent cell stress and inflammation or microbial insults) has been linked to the very high mutational burden in typical driver genes of RDEB‐associated SCC (including *TP53, HRAS, NOTCH1 and CDKN2A*).^[^ ^68^ ^]^ Another anti‐neoplastic therapeutic target is miR‐10b, a potential pro‐metastatic microRNA conferring cancer stem cell‐like properties that is upregulated in EB‐SCCs. miR‐10b expression might also prove sensible as a biomarker, to facilitate early and less invasive detection of SCCs in EB, which is often delayed due to their predominant emergence within chronic wounds.^[^ ^108^ ^]^ Recently, Zauner et al demonstrated the feasibility of circulating miRNA signatures in serum samples to be analysed for tumor prediction (RDEB‐SCC).^[^ ^16^ ^]^

## 9 LOOKING BEYOND: CONCEPTUALIZING EB AS A BARRIER‐DISRUPTION DISORDER THAT CAN BE TARGETED BY THERAPEUTIC IMMUNE MODULATION

One of the most important functions of the skin is the formation of a barrier against the external environment, hence providing protection against pathogenic invasion, chemical or physical assaults and unregulated loss of water and solutes. Blisters, erosions and denuded areas are clinical hallmarks in EB and, likewise reflect the extent of disruption of the mucocutaneous barrier.

**FIGURE 1:**
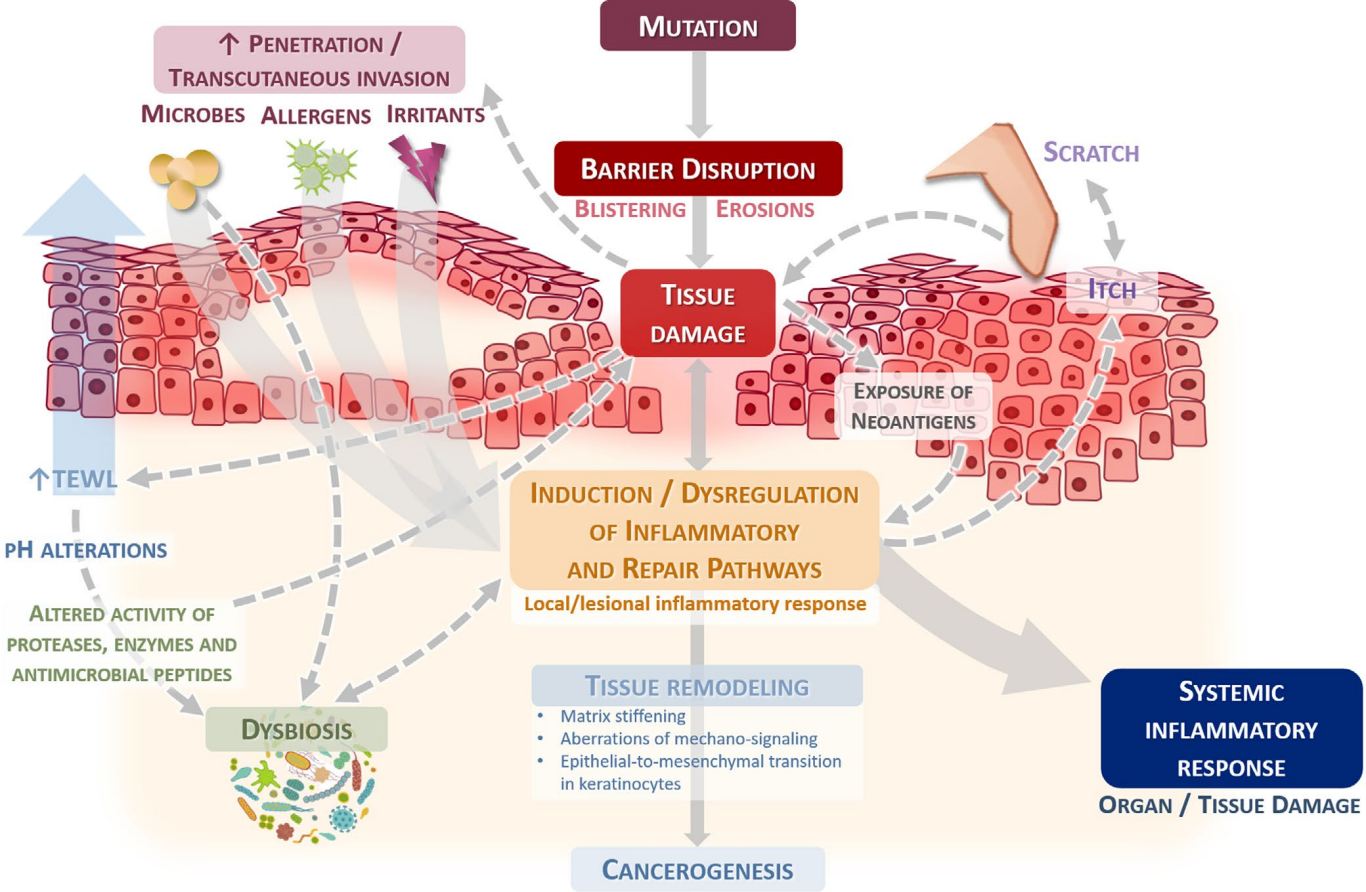
Epidermolysis bullosa is characterized by a genetically determined barrier disruption with chronic tissue damage. This is implicated to favour induction, perpetuation and dysregulation of pro‐ and autoinflammatory responses, causing microenvironmental alterations, dysbiosis and tumorigenic tissue remodelling

The genetically determined barrier disruption may facilitate transcutaneous invasion of irritants, microbes and allergens, which additionally contribute to the perpetuation of pro‐ and autoinflammatory responses, further driving microenvironmental alterations, dysbiosis and tumorigenic tissue remodelling.^[^ ^109^ ^]^ (Figure 1) Leakage and increase of skin permeability/penetrability and inflammatory sequelae may foster itch, followed by scratching and advanced barrier disruption, as well as trans‐epidermal water loss with alteration of skin pH value and thus activity of proteases, enzymes and antimicrobial peptides (AMPs). This helps maintain a chronic, destructive and unproductive immune response that favours tissue and organ damage.^[^ ^110^, ^111^ Moreover, permanent regeneration efforts to drive tissue repair likely contribute to exhaustion of the skin stem cell pool, thereby fostering chronic, non‐healing wounding.^[^ ^112^ ^]^

While in localized EB variants inflammatory aberrations mainly affect the micromilieu of lesional skin, a systemic inflammatory response in severe subtypes such as RDEB contributes to the extracutaneous morbidity of EB.^[^ ^1^, ^18^, ^19^, ^20^, ^21^ However, inter‐ and intra‐individual variability (consistency) of immune responses, including their main effectors remain to be determined. Individual inflammatory signatures most likely depend on various factors such as distinct molecular aberration, anatomic site, microbial burden, transcutaneous sensitization or autoimmunity. Thus, the (individual) immune modalities necessary to support rational translation into efficient, safe, feasible, and tolerable therapies, are currently rather ill‐defined.

Against this background, immunological traits nevertheless define new targets for therapy, aimed at skin barrier restoration, infection control/surveillance of microbial burden/dysbiosis, immune response/immune modulation, anti‐neoplastic interference and impairment of epigenetic drivers of the disease. Various modalities have been or are currently evaluated for their translational potential as briefly outline above. In this context, direct targeting of defined and known disease subtype‐related components, for example with small molecules or monoclonal antibodies, is considered more specific and potentially safer and more efficient. Moreover, repurposing of drugs in stock or pipeline for (more common) immune and neoplastic diseases, including atopic dermatitis as a prototypic barrier disorder, hold some promising potential to enlarge and personalize our therapeutic armamentarium in EB.

## CONFLICTS OF INTEREST

The authors declare that they have no conflicts of interest.

Research data are not shared.

## AUTHOR CONTRIBUTIONS

CP and ML wrote the paper in consultation with JB. All authors have read and approved the final manuscript.
